# Elicitor-Induced VOC Emission by Grapevine Leaves: Characterisation in the Vineyard

**DOI:** 10.3390/molecules27186028

**Published:** 2022-09-15

**Authors:** Christelle Lemaitre-Guillier, Agnès Chartier, Christelle Dufresne, Antonin Douillet, Stéphanie Cluzet, Josep Valls, Nicolas Aveline, Xavier Daire, Marielle Adrian

**Affiliations:** 1 Agroécologie, CNRS, INRAE, Institut Agro, Univ. Bourgogne, Univ. Bourgogne Franche-Comté, 21000 Dijon, France; 2 Institut de Chimie Organique et Analytique, ICOA, UMR 7311, Université d’Orléans, CEDEX 2, 45067 Orléans, France; 3 Institut Français de la Vigne et du Vin (IFV), 33290 Blanquefort, France; 4 Univ. Bordeaux, INRAE, Bordeaux INP, Bordeaux Sciences Agro, OENO, UMR 1366, Equipe Molécules d’Intérêt Biologique (MIB), ISVV, 33140 Villenave d’Ornon, France

**Keywords:** volatile organic compounds (VOCs), grapevine, elicitor, vineyard, stir bar sorptive extraction (SBSE)

## Abstract

The present study is aimed at determining whether leaf volatile organic compounds (VOCs) are good markers of the grapevine response to defence elicitors in the field. It was carried out in two distinct French vineyards (Burgundy and Bordeaux) over 3 years. The commercial elicitor Bastid^®^ (Syngenta, Saint-Sauveur, France) (COS-OGA) was first used to optimise the VOCs’ capture in the field; by bagging stems together with a stir bar sorptive extraction (SBSE) sensor. Three elicitors (Bastid^®^, copper sulphate and methyl jasmonate) were assessed at three phenological stages of the grapevines by monitoring stilbene phytoalexins and VOCs. Stilbene production was low and variable between treatments and phenological stages. VOCs—particularly terpenes—were induced by all elicitors. However, the response profiles depended on the type of elicitor, the phenological stage and the vineyard, and no sole common VOC was found. The levels of VOC emissions discriminated between weak (Bastid^®^ and copper sulphate) and strong (methyl jasmonate) inducers. Ocimene isomers were constitutively present in the overall blends of the vineyards and increased by the elicitors’ treatments, whilst other VOCs were newly released throughout the growing seasons. Nonetheless, the plant development and climate factors undoubtedly influenced the release and profiles of the leaf VOCs.

## 1. Introduction

Elicitors are well-known compounds that induce plant defences and are employed to enhance resistance to pathogens. They cover various chemical classes, including lipids, peptides, proteins and carbohydrates. Some of them originate from micro-organisms or plants, and are referred to as microbe- or damage-associated molecular patterns (MAMPs and DAMPs), respectively. They are specifically perceived as danger signals by plant receptors [1,2]. Elicitor perception triggers a cascade of signalling events that usually lead to the production of defence compounds such as phytoalexins or pathogenesis-related (PR) proteins [3]. Phytohormones, such as salicylic acid or jasmonic acid, are also involved in plant defence signalling downstream of MAMP/DAMP perception [4,5]. Their functional analogues—among which are acibenzolar-S-methyl (BTH) and methyl jasmonate (MeJA)—can elicit plant defence reactions and induce resistance to pathogens [6,7,8]. In addition to biological compounds, abiogenic substances, including heavy metals such as mercury or copper salts, have been shown to be defence elicitors [9,10,11,12].

Elicitor-induced resistance to pathogens represents an attractive strategy to reduce the use of synthetic pesticides required to control plant diseases. This is particularly true for the grapevine (*Vitis vinifera*), an economically important crop grown in many regions of the world. The grapevine is highly susceptible to powdery mildew and downy mildew caused by the ascomycete *Erysiphe necator* and the oomycete *Plasmopara viticola*. Controlling these diseases usually requires more than ten pesticide applications per year. The oligosaccharidic elicitor Bastid^®^ was registered in France in 2016 for control of the grape powdery and downy mildews [13]. However, a number of vineyard trials in the past few years led to the conclusion that the efficacy of this product was quite variable [14] and limited its use. More generally, the use of elicitors still raises many unanswered questions. Dedicated outdoor studies are needed to determine (*i*) to what extent grapevine defences are induced following elicitor application (time course and level of induction) and (*ii*) how long the protection against diseases lasts. A number of factors can indeed influence the plant’s responsiveness to elicitors, such as the genotype, the plant development stage, leaf age [15], abiotic stresses (climate) and nutrition [16]. Knowledge of the factors that influence elicitation is largely incomplete. Monitoring the grapevine defence responses in the vineyard is needed to address these questions. Defence metabolites are good candidates as markers of effective elicitation since they are end-products that are active against bioagressors and amenable to automated high-throughput measurement. Among them are the well-studied stilbene phytoalexins associated with resistance to downy mildew and grey mould [17,18,19]. They are induced by various elicitors [6,8,12,15,20,21]. However, their analysis requires destructive sampling and is time-consuming because of several extraction and analysis steps. VOCs are other defence compounds that are currently attracting growing attention. These metabolites play an important role in the adaptation of plants to their environment by attracting pollinators or repelling pests, and triggering resistance to abiotic and biotic stresses [22,23,24,25]. Regarding plant–pathogen interactions, VOCs can indirectly act as airborne signals that prime the defence reactions against pathogens in neighbouring plants [26,27,28], or directly as antimicrobial compounds [29,30].

Previous works have shown that elicitors can trigger VOC emission in plants [21,27,31,32,33]. In greenhouse conditions, application of the elicitors, sulphated laminarin, chitosan (a chito-oligosaccharide (COS)) and Bastid^®^, on grapevine plants increased the emission of mono- and sesquiterpenes [21,34]. The elicitor Bastid^®^ is composed of a mixture of oligochitosan and oligogalacturonic acid (COS-OGA) complexed by cations, which act synergistically in Arabidopsis [35]. It induces the emission of mono- and sesquiterpenes in tomatoes [31], rice [32] and grapevines [21], and also has a potential antimicrobial action [36]. We also used copper sulphate (CuSO_4_) as an abiotic elicitor. It has been employed as a fungicide in a Bordeaux mixture for controlling grapevine downy mildew since the end of the 19th century. It can elicit the accumulation of stilbene phytoalexins in grapevines [12] and the emission of various VOCs, including terpenes in tomatoes [31]. Methyl jasmonate (MeJA) is a phytohormone involved in many physiological processes including terpene biosynthesis. Besides this role, MeJA triggers production of volatile terpenes in grapevines [37] and induces resistance against plant diseases such as grapevine powdery mildew [6] and downy mildew [8]. Several techniques of plant VOC sampling are now described (see [38] for a review), including SBSE previously used in a greenhouse study [21]. The same approach was used to characterise elicitor-induced VOC profiles in the field and to determine whether VOCs are good candidates as markers of the grapevines’ response to elicitors in the vineyard. We selected the optimal VOC collection method, and then analysed the VOCs induced by different elicitors applied across the growing season. The experiments were set up over three growing seasons (2017–2019), in the two highly different Bordeaux (cv. Cabernet franc) and Burgundy (cv. Chardonnay) French vineyards. Three elicitors were assessed: Bastid^®^, MeJA and CuSO_4_ at three phenological stages of grapevine. Bastid^®^ was chosen as the common elicitor for both of the sites because it is registered as an elicitor and marketed. The other two elicitors were site-dependent: each site used the elicitor already tested in field conditions. In parallel to VOCs’ analysis, the most abundant polyphenols contained in grapevines and those known to be involved in plant defence (i.e., stilbenes) were quantified. The influence of local environmental factors on VOCs detection was also considered.

## 2. Results

The 2017 and 2018 experiments were dedicated to methodological development. The year 2019 was considered as the actual analysis for VOCs response to elicitors since the experimental conditions were settled and no major stress—whether biotic or abiotic—was noticed. Consequently, retroactive comparisons with the 2018 data were made to corroborate the findings of 2019.

### Polyphenol Analysis

**Figure 1:**
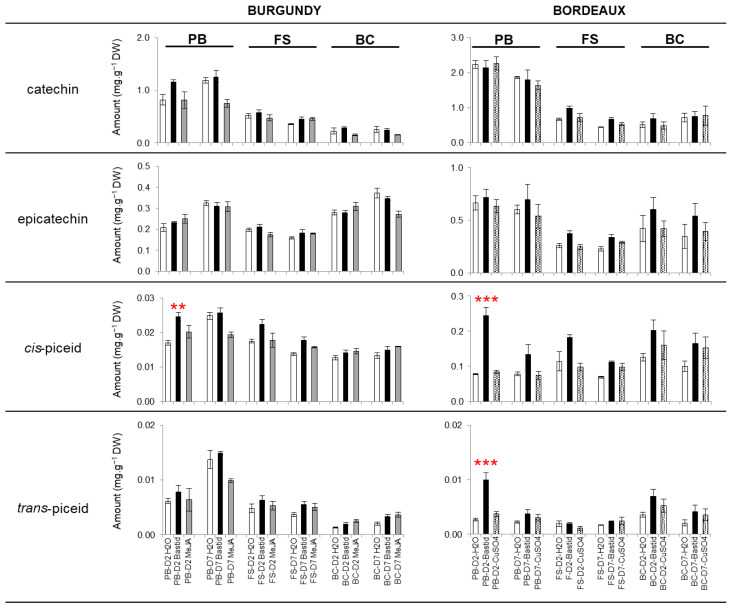
Quantification of phenolics in elicitor-treated grapevine leaves. Experiments were performed in Burgundy (cv. Chardonnay) and Bordeaux (cv. Cabernet franc) vineyards at three phenological stages of grapevine in 2019. Treatments: H_2_O (control) (white), Bastid^®^ (black), MeJA (grey) and CuSO_4_ (dotted grey). Catechin, epicatechin, *cis*- and *trans*-piceids were identified and quantified by LC-MS analysis. Concentrations were averaged from 3 replicates and adjusted to the dry weight (DW) of green leaf powder. Phenological stages: pre-blossom: PB, fruit-set: FS and bunch-closure: BC. D2 and D7: samples collected at two- and seven-days post treatment, respectively. Significance *p* value: 0 < *** < 0.001 < ** < 0.01.

The elicitor treatments were compared for their capacity to induce phenolic production, with a special focus on stilbene phytoalexins, a well-known plant defence response compound. Stilbenes and other phenolics were monitored along three phenological stages (pre-blossom, PB; fruit-set, FS and bunch-closure: BC) two- and seven-days post treatment (D2, D7) (detailed in the Section 7 and Table S1 (Supplementary Materials)). The flavonoids catechin and epicatechin were the most accumulated phenolics in both of the vineyards in 2019, but were not particularly induced by the elicitors (Figure 1).

Among the stilbenes, only the piceids were significantly induced by Bastid^®^, at PB-D2 (*cis*-piceid in the two vineyards, and *trans*-piceid in Bordeaux vineyard). MeJA and CuSO_4_ did not induce those stilbenes significantly. In 2017 and 2018, the catechin and epicatechin were accumulated the most and in similar amounts, regardless of Bastid^®^ treatment or the phenological stage of grapevine. The *cis*- and *trans*-piceids were also significantly accumulated in the Bastid^®^-treated leaves and more so in the Bordeaux vineyard (Figure S1). 

*Stilbene production was not induced by elicitors, except for Bastid^®^ on D2*.

## 3. VOC Emission Analysis

### 3.1. Determination of the Optimal Sampling Method

Several conditions of VOC collection were assessed and large amounts of data were generated (detailed in the Section 7 and Table S2). To validate our approach for measuring VOCs in the field, we first compared the profiles of the VOCs surrounding the grapevine foliage with those at the edge of the plot (Ext) in 2017 and 2018. A principal component analysis (PCA) of the data collected during the growing season showed specific grapevine VOCs emitted by the foliage whatever the treatment (control, elicitor) (Figure S2).

**Figure 2:**
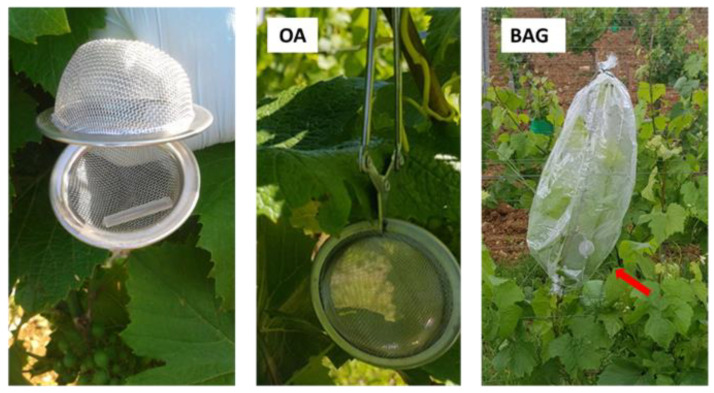
Devices and modes of VOC collection. One SBSE Twister™ was entrapped in a tea ball (left picture), and the device was placed either in the foliage (open-air: OA mode) (middle picture) or enclosed in a bag clipped on a vine shoot (BAG mode) (red arrow, right picture).

VOCs are emitted as a mixture and diluted in the canopy atmosphere. Therefore, technical optimisation was necessary to capture them. Three methods of passive VOC collection using SBSE Twister™ in a tea ball were assessed: OA, OA-CUMUL and BAG (Figure 2).

**Figure 3:**
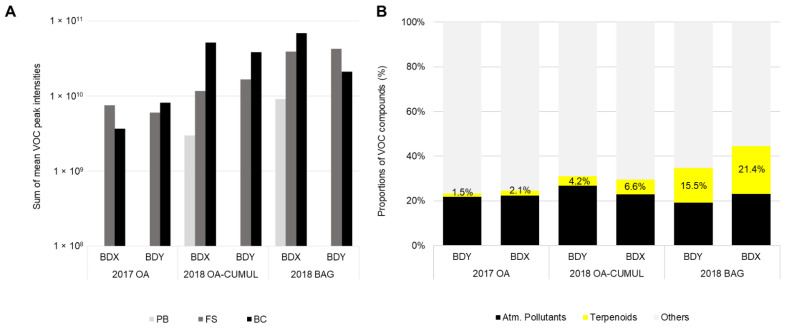
Intensity and diversity of VOCs collected by three collection methods. Three different modes of VOC collection—open-air (OA), open-air cumulated (OA-CUMUL) or bagged (BAG)—were compared in 2017 and 2018 in two vineyards (Burgundy, cv. Chardonnay, Bordeaux, cv. Cabernet franc). (**A**) Sums of the peak area intensities calculated from H_2_O-treated grapevines at three phenological stages (pre-blossom: PB; fruit-set: FS and bunch-closure: BC). (**B**) Distribution of the 100 most counted elemental formulas recorded from Bastid^®^- and H_2_O-treated vines. Vineyards: Bordeaux: BDX; Burgundy: BDY.

The assays performed in both of the vineyards in 2017 and 2018, indicated that the OA mode captured the lowest intensities and proportions of compounds of interest (terpenoids) (Figure 3A,B). The compounds qualified as atmospheric pollutants (e.g., hydrocarbons, heavy metal-containing pollutants) represented around 20% of the captured VOCs while the terpenoids released by the plants only amounted to 1.5 to 2%. When the SBSE sensors were left from three to seven days (OA-CUMUL mode), the intensities increased and the proportions of terpenoids were two–three times higher (ca. 4 to 6% of total VOCs). Only the use of the bags increased both the intensities and the proportions of terpenoids which reached about 15 and 21% for the Burgundy and Bordeaux vineyards, respectively. Therefore, the BAG mode was chosen to collect VOCs in 2019.

The BAG mode was a fast and good compromise between the signal intensity and the number of detected VOCs. It also provided optimal enrichment in the VOCs of interest, i.e., the terpenoids that we particularly focused on. Thus, the BAG mode seemed the most suitable to highlight significant elicitor-induced VOCs and compare VOC emissions in response to the different treatments.

As explained above, the BAG mode of collection was chosen to analyse the effect of Bastid^®^ on the emission of total VOCs (all of the detected ones) and terpenes in 2018 and 2019. In order to determine if the VOC emission was induced by an elicitor, intensities were compared (fold change, FC) to those detected in the control water. The averaged FC of compound per elicitor was calculated against H_2_O-treated grapevines (control) values. Overall, the FCs were calculated throughout the growing season and demonstrated some fluctuations associated with phenological stages (for example, for the terpenoids ranging from 0.90–1.59 in Burgundy and 0.91–1.53 in Bordeaux (detailed in Section 7 and Figure S6). Because of these variations, the FC threshold was set to 1, above which ratio was considered as an induction (Table 1). Although the Bastid^®^ treatment did not really trigger an increase in the total VOCs, the emission of terpenoids was only slightly enhanced (FC~1.25) in both vineyards, only in 2019. It was noteworthy that the vineyards suffered from severe diseases that affected plant health in 2018, and consequently exacerbated the basal defence (H_2_O control) used as a reference to calculate ratios. 

### 3.2. Bastid^®^-Induced VOCs 

The 2019 dataset was used to select Bastid^®^-induced VOCs when fit to the following parameters; i.e., when FC > 1 (Bastid^®^ vs. H_2_O-treatment calculated at each time point of each phenological stage) was observed for at least half of the sample sets. From this, the lists composed of 15 and 11 Bastid^®^-induced VOCs were obtained for the Burgundy and Bordeaux vineyards, respectively (Table 2). Among them, 9/15 and 7/11 (ca. 60%) were terpenoids. They were mainly monoterpenes, including pinenes (α- and β-pinenes, α- and γ-terpinene), and ocimenes isomers (*cis*-β-, *trans*-β- and allo-ocimenes). Only *EZ*-allo-ocimene was in common between the two lists, but not systematically induced at all stages. Some of the listed compounds were already induced in the 2018-BAG experiment, e.g., α- and β-pinenes, *EZ*-allo-ocimene and (*E*)-4,8-dimethylnona-1,3,7-triene (DMNT). Overall, the Bastid^®^-FC means (FC_m_) from all of the compounds of each list were 1.3 and 1.8 for the Burgundy and Bordeaux vineyards, respectively.

### 3.3. MeJA- and Copper Sulphate-Induced VOCs 

As previously described for Bastid^®^, the VOCs induced by MeJA and CuSO_4_ were listed (2019 data). Twenty-eight VOCs were induced (FC > 1) in at least 50% of the MeJA-treated grapevines, and 22 in the CuSO_4_-treated grapevines (Table 3). Terpenoids represented 67% and 68% of the VOCs induced by MeJA and CuSO_4_, respectively. MeJA and CuSO_4_ samples shared α- and β-pinenes, *cis*-β-, *EZ*-allo-, *EE*-allo- and *trans*-β-ocimenes, α-copaene, isocaryophyllene and β-caryophyllene, limonene, humulene and DMNT as induced VOCs. Overall, the FC_m_ calculated from all of the compounds of each list were 4.8 for MeJA and 1.6 for CuSO_4_.

*The three elicitors induced VOCs with emission profiles depending on the time point of sampling, the vineyard and the elicitor. The most common elicitor-induced VOCs wereα- andβ-pinenes,* cis-*β-, trans-β-, EE- and EZ-allo-ocimenes, limonene, isocaryophyllene and DMNT. Bastid^®^ and CuSO_4_ (ca. 1 < FCm < 2) were weak VOC inducers, whereas MeJA was a stronger one (FCm > 4)*.

### 3.4. Vineyard Blends Characterisation

The results presented above demonstrate that the grapevines’ response to elicitors cannot be measured by analysing a single VOC. A panel of VOCs has to be analysed, and VOCs are not systematically detected in all conditions. VOCs are emitted in response to multiple stresses, so that the detection of the elicitor-induced VOCs among all of the detected odours can be a real challenge. Therefore, we analysed the data in a different manner by considering the 10 most intensely detected VOCs (called TOP10 hereafter) arbitrarily considered responsible for the blend of the vineyard plot after treatment. Some compounds could be considered exogenous, unlikely to be emitted by vines but rather environmental and/or experimental contaminants and so were considered for data analysis but not as putative markers. For each data set obtained in 2019, the TOP10 lists were drawn up for the Burgundy and Bordeaux vineyards, and the VOCs were compared and ranked according to their amounts. Then, the TOP10 lists were compared with the H_2_O lists (Table 4 and Table 5). 

When compared to the H_2_O control, elicitor induced more new compounds in the Burgundy than in Bordeaux vineyard (Venn diagrams). The TOP10 VOCs of the H_2_O control, considered as components of the basal blend of the plot, were almost similar in both vineyards across the three phenological stages (even if their respective ranking differed), irrespective of the cultivar and the environment. Apart from a few variants, they both constitutively contained *cis*-β-ocimene, oxime-methoxy-phenyl (to a large extent), nonanal, 3- and 4-hexen-ol acetate, and acetophenone. The *cis*-β-ocimene ranked first only once. It was detected together with other ocimene isomers (*trans*-β-, *EZ*- and *EE*-allo-ocimenes), lower in the TOP10 list, especially at the late phenological stages of grapevines. In the Burgundy vineyard, other terpenes such as α-copaene, β-caryophyllene were detected in the basal blend. 

The TOP10 elicitor-induced VOCs were characterised by the global enhancement of ocimene emission. Concomitantly, other VOCs such as nonanal or β-caryophyllene went up in the ranking list, or even reached the pole position. The impact of elicitor treatment on the TOP10 ranking was higher in the Burgundy than in the Bordeaux vineyard with more modifications (Table 4 and Table 5). In the MeJA-elicited samples, β-caryophyllene, limonene, bicyclo [5.2.0]nonane 2-methylene-4,8,8-trimethyl-4-vinyl-, β-bourbonene, humulene and DMNT emissions were enhanced and moved up in the rankings. In the Bastid^®^ and CuSO_4_-treated plots of the Bordeaux vineyard, 1,3-benzenedicarboxylic acid 5-(dimethylamino) was increased however it was unlikely to be emitted by the plants but rather present as a local pollutant.

**Figure 4:**
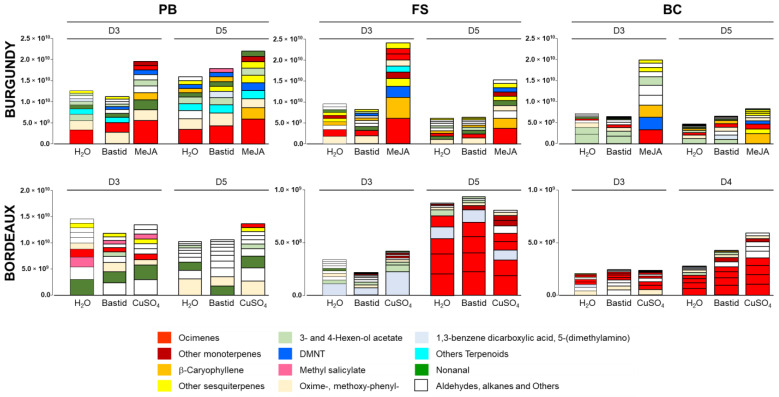
Intensities of the TOP10 VOCs. Peak area intensity values of the TOP10 VOCs detected in Burgundy (cv. Chardonnay) and Bordeaux (cv. Cabernet franc) vineyards in 2019 on D3 and D5 (three- and five-days post treatment) at three phenological stages of grapevine: pre-blossom: PB; fruit-set: FS and bunch-closure: BC. Grapevines were treated with H_2_O (control) or the elicitors Bastid^®^, MeJA and CuSO_4_.

The intensity of the TOP10 VOCs globally decreased from the PB to the BC stage in both vineyards (Figure 4). In Burgundy, a mixture of ocimenes was mostly enhanced at the three stages independently of the treatment, with a noticeable decrease at the BC stage. At this later stage, the green-leaf volatiles (GLV) 3- and 4-hexen-ol acetate were the most released VOCs in the control and Bastid^®^-treated grapevines, whereas the ocimenes decreased. DMNT was only detected in Burgundy, at all stages and particularly in response to MeJA. MeJA treatment consistently induced high accumulation of the TOP10 VOCs, preferentially at the FS stage, while monoterpenes (ocimenes) and sesquiterpenes (especially β-caryophyllene) were enhanced. In the Bordeaux vineyard, the TOP10 VOCs were 20-fold reduced at the FS and BC stages in comparison to the PB stage. The VOC blends following Bastid^®^ and CuSO_4_ treatments were often equivalent to the basal H_2_O blend. Unlike in Burgundy, noticeable amounts of methyl salicylate, alkanes and aldehydes (including nonanal) were measured at the BP-D3 time point. At the FS and BC stages, the blend was mostly composed of monoterpene ocimenes (especially at the FS stage).

*The basal blend slightly varied in nature and intensity depending on the study site and the time of collection. VOCs globally decreased along seasonal growth, in both of the vineyards. Induced VOCs were more detectable in the plot blend when a strong elicitor was used*.

### 3.5. Impact of Environmental Factors on VOC Detection

**Figure 5:**
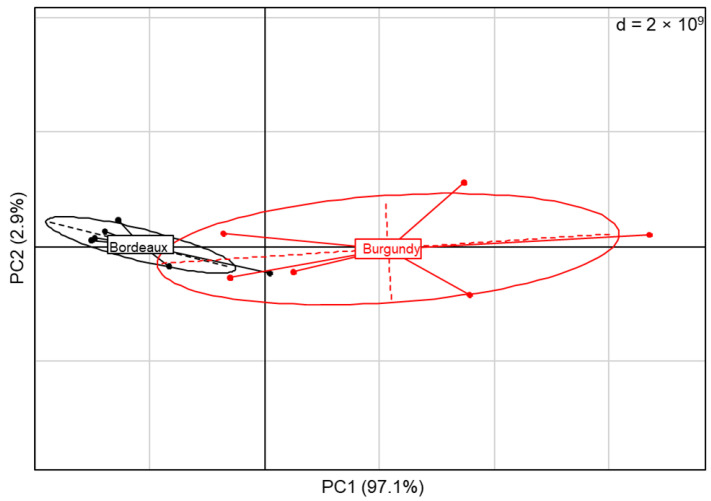
Principal component analysis (PCA) of total terpenes emission in the vineyards in 2019. Values correspond to the sum of mono- or sesquiterpenes detected in response to Bastid^®^ treatment at two time points and at three phenological stages of grapevines in Burgundy (cv. Chardonnay) and Bordeaux (cv. Cabernet franc) vineyards.

The PCA showed that the Bastid^®^-induced VOCs clearly distinguished terpene emissions by the two vineyards in 2019 (Figure 5). Regardless of the differences observed between treatments in both of the sites, VOC emission could depend on the environmental conditions of the grapevines.

**Figure 6:**
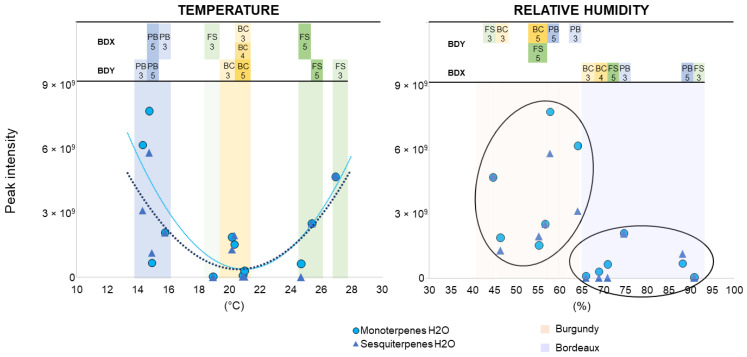
Impact of climate parameters on terpene detection on H_2_O-treated grapevines. Grapevine terpenes were analysed three- and/or five-days post treatment with H_2_O (control). Values for terpenes correspond to peak area values from Burgundy (cv. Chardonnay) and Bordeaux (cv. Cabernet franc) vineyards in 2019. Collection days and phenological stages of grapevines (pre-blossom: PB; fruit-set: FS, bunch-closure: BC) are indicated above the climate parameters for each vineyard (Burgundy: BDY; Bordeaux: BDX). The shapes of the tendency curves were chosen to best fit the point values.

**Figure 7:**
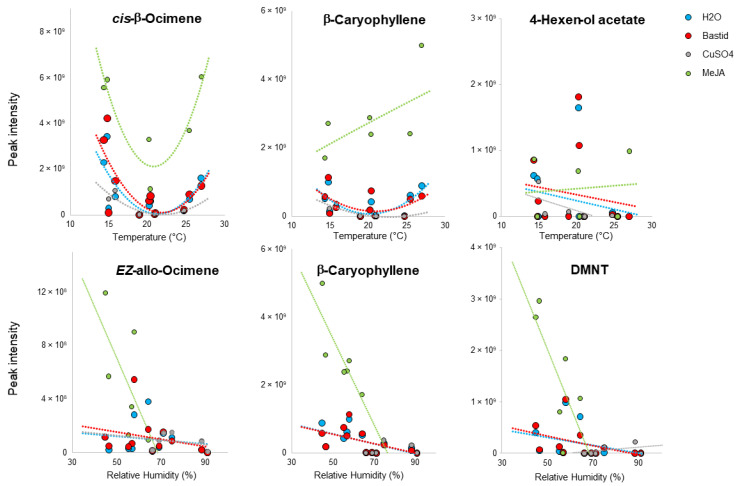
Impact of mean temperature and relative humidity on the detection of targeted monoterpenes, sesquiterpenes and “Other VOCs” induced by elicitors. The graphs were built from 2019 data. Grapevine emission of terpenes analysed three- and five-days post treatment with H_2_O (control) or an elicitor of plant defence (Bastid*^®^*, MeJA or CuSO_4_) at three phenological stages of grapevines (pre-blossom, fruit-set, bunch-closure) in Burgundy (cv. Chardonnay) and Bordeaux (cv. Cabernet franc) vineyards. Values correspond to VOC peak intensities and the shapes of the tendency curves were chosen to best fit the point values.

Among the factors likely to explain these differences, our experimental conditions allowed us to explore two of them, of climatic origin (mean temperature and relative humidity on the day of collection). The impact of climate was first evaluated on the terpenes (mono- and sesquiterpenes) most emitted by the H_2_O-treated grapevines (Figure 6), and then on those emitted by the elicited grapevines (Figure 7). In 2019, the mean temperature values of the sampling days overlapped and ranged from 14 °C to 27 °C in the Burgundy and Bordeaux plots. However, relative humidity was lower in Burgundy (44–56%) than in Bordeaux (66–90%).

In response to the temperature variations, the Burgundy and Bordeaux vineyards exhibited similar U-shaped curves of terpenes emissions, with a minimum at 20 °C. The effect of relative humidity was rather different, with VOCs clustered according to the vineyards. The TOP10 monoterpenes, sesquiterpenes and “Other VOCs” detected in response to all of the treatments were assessed in the same manner for the two vineyards. The detections of representative or major compounds are illustrated in Figure 7, while overall analysis of terpenes (mono- and sesquiterpenes) and “Other VOCs” are presented in Figures S3–S5.

All of the monoterpenes adopted a U-shape curve vs. temperature (Figure 7 and Figure S3), while differences were observed for sesquiterpenes and “Other VOCs” (Figure 7 and Figures S4 and S5). Interestingly, the strong induction of sesquiterpenes, such as β-caryophyllene, bicyclo[5.2.0]nonane and humulene emissions by MeJA, increased with temperature (Figure S4). On the other hand, the detection of those VOCs dropped with increasing relative humidity, especially after MeJA treatment. Among the “Other VOCs” group, 4-hexen-ol acetate (Figure 7) and 1,3-benzenedicarboxylic acid, 5-(dimethylamino) (Figure S5) were detected independently of temperature. Furthermore, MeJA-induced DMNT detection seemed independent of rising temperature and decreased with relative humidity (Figure 7).

*Climate parameters (temperature and relative humidity) seem to have an impact on VOCs detection depending on their nature and the strength of the elicitor*.

## 4. Discussion

### Elicitation of Polyphenols

Polyphenols are well-known to be involved in grapevines’ defence. We analysed the production of polyphenols, especially of the stilbene phytoalexins, to check whether the elicitors used in the study efficiently stimulated such responses in field conditions. Over the 3 years, Bastid^®^ and CuSO_4_ mainly induced the accumulation of the two flavonoids catechin and epicatechin, and also *cis*- and *trans*-piceids, but at different time points. Both piceid isomers had accumulated after Bastid^®^ treatment in greenhouse conditions [20]. Unexpectedly, neither resveratrol nor viniferins were significantly induced, even when MeJA was used. As a result, MeJA had hardly any effect even though it had been reported as a stilbene inducer [6,8,39,40,41]. However, we used a lower concentration of MeJA (2.5 mM) compared to the aforementioned studies. Goufo, et al. [42] indicated that the leaves, exposed to environmental stresses, had a low concentration of the main stilbenes (*trans*-ε-viniferin, *trans*-resveratrol). In our study, this can be explained by the fact that the sampling points were rather limited (only two) and maybe too late (two and seven days after the last elicitor treatment) to correspond to maximum stilbene accumulation. Nevertheless, the accumulation of piceids in response to Bastid^®^ was higher in the Bordeaux plot planted with cv. Cabernet franc than in the Burgundy plot planted with cv. Chardonnay. This could suggest a cultivar effect since stilbene synthesis is genotype-dependent [43,44].

## 5. Elicitation of VOC Emission

### 5.1. Development of a Method for VOC Collection in the Field

Our initial aim was to develop a user-friendly and non-destructive method for monitoring VOC emissions in the vineyard. Following Lemaitre–Guillier, et al. [21], VOCs were collected using an SBSE device that allows for higher sensitivity and sorption capacity, especially for sesquiterpene compounds [45], in comparison to the solid-phase micro extraction technique (SPME) employed by Chalal, et al. [34]. This method was used on detached berries [46]. In the field, SBSE was used in tea ball assays by Cheung, et al. [47] to detect the infection of citrus trees by the Tristeza virus, but also by Kfoury, et al. [48] in direct contact on tea leaves challenged to herbivory attack. We chose passive collection using an SBSE sensor in a tea ball left in open air (OA mode) as a first attempt in 2017. This approach enabled us to detect a great number of VOCs, but with poor signal intensities and many of them were probable atmospheric pollutants commonly originating from road transport [49]. An improvement was attempted by leaving the sensors longer in the foliage, up to 7 days (OA-CUMUL mode). This second approach increased the signal of the VOCs of interest but still detected diverse VOCs unrelated to an elicitation response. Then we moved to SBSE sampling by confining the sensor together with the vine shoot in a bag for 4 h (BAG mode). This procedure deriving from that described by [34] was firstly and partially employed in 2018 and confirmed in the 2019 growing seasons. It resulted in a ca. five-fold signal increase with a strong enrichment in terpenes compared to the OA mode. This improvement was undoubtedly attributable to a concentration of these leaf-emitted compounds in the bag atmosphere. The BAG method can be criticised because it increases temperature and humidity [50], but all of the comparisons were made versus a control under the same times and experimental conditions. This method has the advantage of using SBSE under a static mode since the bagging time is limited to avoid the release of artefactual stress-related compounds. To our knowledge, this has not been previously described for field VOC sampling in the vineyard and may be used as a non-invasive surveillance system for warning of plant disease by detecting specific or universal VOC fingerprints [51].

### 5.2. Induction of VOC Emission by Elicitor Treatments

VOC emission was variable according to the location and hence the cultivar, the time of collection, and the phenological stages of grapevines that made it difficult to assign the elicitation response to recurring VOC emissions. Based on the FC_m_ values, Bastid^®^ and CuSO_4_ appeared to be weak VOC inducers whereas the plant hormone MeJA was a strong one. To our knowledge, such a study on Bastid^®^- and CuSO_4_-induced VOCs in the vineyard has not been reported, while MeJA is well documented (see [23] for a review). Nevertheless, all three elicitors induced the emission of certain VOCs, mainly monoterpenes such as ocimene and pinenes. The ocimene isomers (β-, *trans* β-, *EZ*- and *EE*-allo-) were systematically enhanced after elicitation and were amongst the most emitted ones in both vineyards. These results obtained with plants grown in their natural habitat agree with previous greenhouse results showing the promoting effect of Bastid^®^ on β-ocimene emissions [21]. The monoterpene β-ocimene is found in many plant species and has multiple functions, including pollinator attraction and defence against herbivores [52]. It is also one of the VOCs over-emitted by grapevines in response to insect attacks [53,54,55] or following treatment with the elicitor, sulphated laminarin [34]. Allo-ocimene activates the defence response of *Arabidopsis thaliana* against *Botrytis cinerea* [56]. It induces defence genes and suppresses fungal penetration and development [26]. Therefore, it is involved in resistance against fungal diseases. Sesquiterpenes—especially β-caryophyllene and α-copaene—were somewhat induced by the strong inducer, MeJA. Together with α-farnesene and β-caryophyllene, they are known to be released after wounding, and also under abiotic stress [51]. An artificial blend partly composed of β-ocimene, α-farnesene and β-caryophyllene was shown to act as a repellent to the wheat weevil (*Sitophilus granarius*) and the confused flour beetle (*Tribolium confusum*) [24,25]. β-caryophyllene is also induced by *Plasmopara viticola* (downy mildew) in grapevines and is directly linked to their resistance against this pathogen [30]. In 2018, the Burgundy and Bordeaux plots underwent severe downy mildew and erinea attacks, and elicitation with Bastid^®^ probably amplified the basal β-caryophyllene release. Some green leaf volatiles (GLVs; 3- and 4-hexen-ol acetate and nonanal) and homoterpene (DMNT) were also recurrently induced by elicitors. Together with ocimenes, GLVs were constitutively detected among the 10 most emitted VOCs in the Burgundy and Bordeaux vineyards. They play a role in the plant response to environmental biotic and abiotic stresses [57,58,59,60]. Hexenyl acetate and DMNT, which are commonly detected in the field VOC background, have been depicted as constituents of an olfactory signature recognised as an attractive specific blend by leafhoppers [55]. Oxime methylphenyl was often the most abundant VOC in our samples. This compound has antioxidant and antimicrobial activity. It has been constitutively detected in apple [61] and among the major peaks of *Urtica dioica* leaves [62].

### 5.3. Impact of Climate on VOC Emission

A grapevine plot has its own characteristics (cultivar, rootstock, vine age, soil, farming practices) and is impacted by variable environmental factors at the origin of multiple stresses (climate, diseases) that may influence the composition of the vineyard blend. Plant VOC emissions depend on environmental conditions, and a distinction is made between constitutive and stress-induced VOCs [63]. Regardless of the TOP10 compounds emitted in both vineyards, VOC emissions decreased along the 2019 growing season, from the PB to the BC stages, whatever the treatment. This decrease could be the consequence of plant development and aging. Literature on the correlations between VOC emission and ontogeny is scarce. Research on trees (*Betula p.*, *Populus t.* and *Sambucus n.*) indicated both the total volatiles and individual compounds (among which (*Z*)-3-hexenyl acetate, (*Z*)-3-hexen-1-ol, (*E*)-β-ocimene, methyl salicylate, and β-caryophyllene) significantly decreased between two seasonal samplings (in June and August) [64]. However, VOCs are admittedly released from the trichomes and stomata so that their functional activities would impact emission rates [65]. The terpenoids’ emission decrease has been described in *Rhododendron tomentosum*, correlated with the density of active glandular trichomes that deteriorate as time passes throughout the season. The capacity of leaves to synthesize and emit VOCs is somewhat associated with young leaves [66]. Climate undoubtedly impacts on VOC emissions and Ju et al. [67] demonstrated that the stomatal density aperture decreased in *Vitis vinifera* grapevines submitted to 15 days of drought. Moreover, VOC composition was modified, with increased concentrations of GLVs, such as 2- and 3-hexenal. However, in this study, we did not observe the same PB > FS, BC profiles in 2017 and 2018, but rather increased PB < FS < BC, that suggests other factors involved in the VOC emissions (Figure 3). Therefore, we studied the potential impact of climate (temperature and relative humidity).

We first used the TOP10 VOCs detected in the control H_2_O-treated grapevines to evaluate the effects of climate factors on VOC emission. Regardless of mean temperature, we observed a U-shape curve of VOC amounts, with minimum emission at around 20 °C. These results are surprising since VOCs emission was thought to be enhanced by increasing temperature [68]. Using a similar bagging approach, the leaf VOC emission rates of tree branches (*Betula p., Populus t.* and *Sambucus n.*) were increased between 16 and 32 °C and plateaued beyond [64]. In apple trees, Vallat, et al. [69] observed that terpenes and nonanal releases were dependent—even at a daily scale—on multiple factors such as temperature, daily rainfall and relative humidity. Mofikoya et al. [66,70] showed that terpene compounds condensed in leaf cuticular waxes at low temperature (ca. 15 °C) and then re-released when temperature increased and such a phenomenon might partly explain the U-shape curve observed in our study. Indeed, from this point of view, a difference is made between emission and detection (i.e., absorption on sensor). Camacho-Coronel, et al. [71] demonstrated that such an accumulation in waxes could last for 15 days while maintaining an inhibitory effect on the germinating fungal spores of the pathogen *Colletotrichum lindemuthianum*. At the early development stage (PB), the Burgundy and Bordeaux vineyards underwent low mean temperatures (14–20 °C) but high rates of VOCs were detected. This could be due to the release of condensed and accumulated VOCs on the leaf surface as a consequence of the rising temperature caused by the BAG collection mode. The temperature undoubtedly increased in the bags when VOC collection took place from 9:00 to 13:00, causing a “greenhouse” effect, and thereby an exaggerated number of VOCs. The second part of the U-shaped profile with an increasing detection rate from 20 °C would reflect the daily emission and direct release of VOCs, whose release increased with ambient temperature [72]. This study also shows the stronger efficacy of MeJA, as compared with Bastid^®^ and CuSO_4_, to induce the VOCs emission whatever the temperature values. Moreover, increased relative humidity had a global negative effect, particularly on the emission of some VOCs (*EZ*-allo-ocimene, β-caryophyllene, humulene and DMNT). As particularly seen under the Bordeaux conditions, this may also suggest a specific vineyard response. This specific behaviour against relative humidity cannot be explained by the stomatal aperture, as the stomata are normally open when humidity is high. As the factors that drive the release of VOCs by stomata remain unclear, our observations on those particular compounds would deserve further attention.

## 6. Conclusions

VOC emission is induced by elicitor treatments in vineyard conditions. Therefore, non-destructive analyses of VOCs are an attractive means of monitoring the plant response to elicitor application. In our study, the basal blends (H_2_O control) emitted by the two vineyards located five hundred kms apart were roughly similar in composition and intensity, which indicates a global constancy. However, we failed to highlight a specific elicitor-induced VOC “biomarker”, most probably because of the weakness of the two elicitors used in this study and the multiple parameters inherent to the fields. Nevertheless, ocimenes seem to be relevant candidate biomarkers, as well as sesquiterpenes (β-caryophyllene). Targeting these specific VOCs or increasing the sensitivity of the detection mode would probably render the analysis more robust. This could be achieved by using an “electronic nose”, or dynamic VOC collection using a portable pump. Further work is needed to better understand the biological significance of VOC emission in field conditions.

## 7. Experimental Section

### 7.1. Experimental Plots

The experiments were conducted in Burgundy and Bordeaux vineyards (France). In Burgundy, they took place in a plot of the vineyard of the University of Burgundy, located in Marsannay la Côte (47°16′45.0″ N 4°58′50.4″ E). It was planted in 2012 with cv. Chardonnay grafted on rootstock 3309C trained in a simple Guyot system, with grapevine plants spaced out by 1 m. In Bordeaux, the experiments took place in the vineyard of Château Dillon at Blanquefort, near Bordeaux (44°55′02.9′′ N 0°38′32.2′′ W), planted with cv. Cabernet Franc trained in a double Guyot system, with grapevine plants spaced out by 1 × 1.5 m. The experiments were performed in 2017, 2018 and 2019. Daily climate parameters (mean temperature, T °C), relative humidity (RH, %) and rainfall (RF, mm) were recorded at the local weather station. The experiments were delimited on four randomised blocks (replicates) of seven plants per treatment. Water (control), elicitor applications and sample collections were performed at different phenological stages of grapevine: pre-blossom (PB), fruit set (FS) and bunch-closure (BC).

### 7.2. Elicitor Treatments

**Figure 8:**
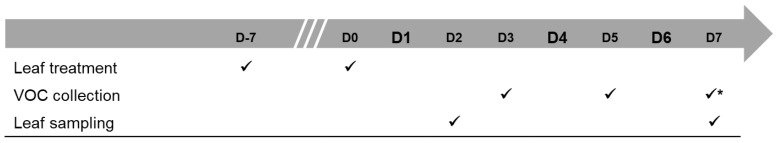
Experimental design. Experiments were conducted in Burgundy (cv. Chardonnay) and Bordeaux (cv. Cabernet Franc) vineyards at three phenological stages of grapevines in 2017, 2018 and 2019. Vines were treated with water (control) or an elicitor twice at seven days interval before the start of the experiment (D0). VOCs were collected on SBSE sensors at 3 time points (D3, D5 and D7). The leaves used for phenolics analyses were picked on D2 and D7. *: not in 2019.

Two elicitor treatments were applied at one-week interval (on D-7 and D0), the second one corresponding to D0 (Figure 8). Fungicide treatments used to protect grapevines against powdery mildew and downy mildew (sulphur and Bordeaux mixture, respectively) were stopped 1 week before the beginning of the experiment. As few experimental handlings differed between the two vineyards, details are indicated when needed. In the Burgundy plot, treatments were executed using a pressure hand sprayer (calibrated at 600 L·ha^−1^), while an SR 420 atomizer (Stihl^®^, Torcy, France, calibrated at 200 L·ha^−1^) was used in the Bordeaux plot.

The elicitor doses were adjusted and are indicated in Table 6. Bastid^®^ was purchased from Syngenta (Saint-Sauveur, France) and used as described by [13]. MeJA was purchased from Sigma Aldrich (Lyon, France). CuSO_4_ was amended as Bordeaux mixture (RSR), containing 20% active copper. Water was used as a control treatment for comparisons.

### 7.3. Phenolics Analysis

The treated or control leaves of the same batches of plants as used for VOC analysis were taken for stilbene analysis (Table S1). Six young leaves (the third or fourth leaves from the apex) were randomly picked in each block on D2 and D7 (Figure 8) and placed in boxes cooled with freezer packs during transportation to the laboratory and then frozen at −70 °C. The leaves were ground to a fine powder in liquid nitrogen prior to freeze-drying. One hundred mg of leaf powder were extracted with 8 mL of methanol at 4 °C overnight. After centrifugation, 6 mL of supernatant was collected, evaporated and the pellet was resuspended in 1 mL of methanol/water (70/30, *v*/*v*). This extract was purified on a Supelclean LC-18 solid phase extraction column (Supelco^®^, Sigma Aldrich, St. Louis, MO, USA). Eluates were evaporated, resuspended in 600 µL of methanol/water (50/50, *v*/*v*), centrifuged (13,500 rpm, 10 min) and stored at −20 °C. Stilbenes were analysed on a 1260 Infinity UPLC (Agilent Technologies, Courtaboeuf, France) coupled to a 6430 triple quadrupole mass spectrometer (Agilent Technologies) as described in Krzyzaniak, et al. [73]. Calibration curves (concentrations ranging from 0.004 to 10 mg·L^−1^) of pure standards were established to determine the concentrations of stilbenes in mg·g^−1^ dry weight (DW) of pure phenolic compounds. Standard stilbenes were purchased from Sigma Aldrich (Lyon, France).

## 8. VOC Collection and Analysis

### 8.1. VOC Collection

Stir bar sorptive extraction (SBSE) Twisters™ (Gerstel GmbH, Co.KG, Mülheim an der Ruhr, Germany), 2-cm long and coated with 1-mm thick polydimethylsiloxane (PDMS) were used as sensors to collect the leaf volatiles [21]. After VOC adsorption, the sensors were kept at room temperature until the entire experiment was over and sent all together for GC-MS analysis.

Three modes of collection were tested to determine optimal VOC collections conditions (Figure 2 and Table S2). For each of them, the Twister™ sensor was placed in a stainless metallic tea ball (called the “device” hereafter (Figure 2)) clipped in the vine foliage. For external controls (Ext), the device was hung on the edges of the plot, about 1 m away from the foliage.

*Open air mode (OA):* the devices were installed on D3, D5 and D7 after the second elicitor treatment (D0) and left exposed in the foliage for 24 h. Three devices (three replicates) were used per condition, separated about 2 m apart inside each block.

*OA cumulative mode (OA-CUMUL):* the devices were all placed in the foliage on D0 and left exposed for 3 days (3D), 5 days (5D) or 7 days (7D). The sensors were picked up on days 3, 5 and 7 after the treatments in each treated block (four replicates).

*BAG mode (BAG):* the upper part of vine stems, consisting of the five to six youngest treated leaves, was wrapped into a commercial oven bag made of polyethylene terephthalate (PET, Albal™, Group Melitta, Minden, Germany) together with the device for 4 h (9:00 a.m.–13:00 p.m.) with four replicates per block. In 2018, the VOCs were collected only on D3 in 2018 in the Burgundy plot.

Over the three 2017—2019 seasons, we accumulated numerous samples (detailed in Table S2). In 2017, to perform experimental optimisation, the VOC samples were collected in open-air mode (OA mode) at three time points post-treatment (D3, D5 and D7). They covered the FS and BC phenological stages, with 94 and 106 samples collected in Burgundy and Bordeaux, respectively.

In 2018, the collection mode was modified to increase the VOC adsorption onto the sensor through accumulation in OA mode by increasing the time of exposure (from 3 to 7 days) or by stacking the VOC emissions in a reduced air volume (BAG mode). Then, 64 and 73 VOC OA-CUMUL samples were obtained for the Burgundy and Bordeaux assays, respectively. The BAG mode was initiated and conducted only on D3 at the FS and BC stages in Burgundy (*n* = 16) and at the three stages in Bordeaux, (*n* = 23). It is important to note that both of the vineyards suffered from severe pathogen attacks in 2018, with successive colonisations by powdery mildew, downy mildew and erinae throughout the plant development. In 2019, 113 and 145 VOC samples were obtained by using the BAG mode in the Burgundy and Bordeaux vineyards, respectively. VOCs were collected on D3 and D5 at the three phenological stages. For practical reasons, some of the sampling days differed from the expected schedule.

### 8.2. GC-MS Analysis

After VOC collection, the Twisters™ were kept at room temperature in their storage vials until analysis. All of the samples were sent to ICOA, (UMR 7311, Orléans, France) for GC-MS analysis. Analyses were carried on an Agilent 7890 Chromatograph hyphenated to a triple quadrupole mass spectrometer (7000C, Agilent Technologies France, Les Ulis, France) equipped with a MPS robotic, thermal desorption unit (TDU 2) (Gerstel, Mülheim, Germany) and a programmed temperature vaporizer inlet (PTV) (Gerstel, Mülheim, Germany), according to [21].

Briefly, desorbed analytes were separated on an RTX-5MS fused-silica capillary column (30 m × 0.25 mm internal diameter; 0.25-mm film thickness; Restek) by increasing GC oven temperature from 30 °C to 250 °C (5 °C·min^−1^) under constant helium flow as a carrier gas (1 mL·min^−1^). The analyte ionisation was performed with an electron ionisation source at 70 eV, maintained at 230 °C. Data were acquired on the MS1 quadrupole held at 150 °C using scan mode. Mass range was *m*/*z* 30 to 350 amu with a scan time of 300 ms. Integration and deconvolution of the acquired data were performed with the Agilent Software Unknowns Analysis (version B.08.00). After deconvolution, the detected peaks were identified against two libraries, the NIST 14 (https://webbook.nist.gov/chemistry/ (accessed on 12 July 2022)) and one constructed in the laboratory. The retention times of the identified peaks were checked against those of a set of standards (Sigma Aldrich, Lyon, France) analysed under the same conditions. Standards used are listed in Table S3. The retention time and the mass spectrum of the standards obtained under these conditions were compared to those obtained after liquid injection in order to check the precision of the home-made library. Quality control of standards (50 µg·L^−1^) was run at the beginning and the end of each analysis. The retention times and the spectrum of identified peaks were checked against those of the standards analysed under the same conditions using, simultaneously, the NIST and the laboratory-constructed libraries. Peaks due to PDMS coating were removed, and only the VOCs identified with a match score > 70 were retained. Terpenoid formulas encompassed C_10_H_16_, C_15_H_24_, C_10_H_16_O, C_10_H_16_O_2_, C_15_H_22_, C_15_H_24_O, C_10_H_18_O, C_10_H_18_O_2_ and atmospheric pollutants were identified from the website list https://www.environnement.gouv.qc.ca/air/cov/liste.html (accessed on 12 July 2022).

### 8.3. Data Treatment

For each year, the effects of the elicitors were examined in phenological periods and at the three time points relative to the H_2_O control sample. In order to build datasets, the lists of compounds associated with their peak area were built according to their time point inside each phenological stage. Principal component analyses (PCAs) were performed from transformed log2 averaged values (RStudio with ade and vegan packages). Phenolics’ amounts from the elicited samples were pairwise compared to the control to detect significantly induced compounds (*p* < 0.05). The values of the replicates of the VOC peak area were averaged per treatment condition, and submitted to comparisons tests. From 2017 and 2018 analysis, the choice of definitive collection mode was made from i- the sums of peak intensities of total VOCs recorded from the H_2_O-treated vines and ii- the Bastid^®^- and H_2_O-treated ones used to evaluate the numbers of terpenoids captured. Significant compounds were determined with Perseus software version 1.6.8.0 (https://maxquant.net/perseus/ (accessed on 12 July 2022)) using ANOVA multivariate statistical analysis (*p* < 0.05) of the time point datasets, and additional *t*-test comparisons (FDR < 0.05) between the elicitor and H_2_O control treatments. In addition to significance criteria, a compound was considered as induced and retained when its intensity was increased compared to the water treatment, meaning its fold-change ratio relative to H_2_O control treatment was over 1 (FC >1) as an arbitrary threshold in at least 50% of the samples per treatment. The results were expressed as total VOC or terpenoid peak area ratio values. As FCs varied along the phenological stage, setting FC > 1 ensured the induction of VOC emissions at all times (Figure S6).

## Figures and Tables

**Table 1:** Bastid^®^ induction levels of total VOCs and terpenoids. Experiments were performed in Burgundy (cv. Chardonnay) and Bordeaux (cv. Cabernet franc) vineyards in 2018 and 2019. VOCs were collected using the BAG mode. Fold changes (FCs) correspond to the averaged of Bastid^®^ over H_2_O (control) treatments ratio values.

Vineyard	Year	Total VOCs	Terpenoids
**Burgundy**	2018	0.69 ± 0.23	0.62 ± 0.13
	2019	1.02 ± 0.25	1.18 ± 0.38
**Bordeaux**	2018	0.78 ± 0.37	0.88 ± 1.26
	2019	1.05 ± 0.48	1.26 ± 0.48

**Table 2:** Bastid^®^-induced VOCs. Bastid^®^-induced VOCs were selected over three phenological stages and two time points (BAG mode), in the Burgundy and Bordeaux vineyards. VOCs were retained when fold change (FC) > 1 at least 4 times out of the 6 time point datasets (ca. > 50% of the collected samples). *: Significant *t*-test (FDR < 0.05) vs. H_2_O control samples. n.i.: not induced. Phenological stages of grapevine: pre-blossom: PB; fruit-set: FS and bunch-closure: BC. Time points: three- and five-days post treatment (D3 and D5). Phenological stages are indicated in the last column when VOCs were induced in the same vineyard in 2018-BAG (FC > 1).

Burgundy		Formula	PB_D3	PB_D5	FS_D3	FS_D5	BC_D3	BC_D5	Induced in 2018
Terpenoids	β-Pinene	C_10_H_16_	1.00	n.i.	1.04	1.07	1.35	1.88	FS
	β-Myrcene	C_10_H_16_	n.i.	1.04	n.i.	2.16	2.28 × 10^7^	3.21	
	α-Terpinene	C_10_H_16_	1.68 *	1.01 × 10^7^	1.76		1.55	1.96 × 10^7^	
	*trans*-β-Ocimene	C_10_H_16_	n.i. *	1.15	n.i.	1.58	2.29 *	1.90	
	*cis*-β-Ocimene	C_10_H_16_	1.43 *	1.23	n.i.	1.33	1.52	1.38	FS, BC
	*E*,*Z* allo-Ocimene	C_10_H_16_	n.i.	1.92 *	1.00	2.25	2.51	1.38	
	Linalool	C_10_H_18_O	1.03	1.22	n.i.	1.21	1.14	1.93	
	1*H*-Cyclopropa[*a*]naphthalene, 1a,2,3,3a,4,5,6,7b-octahydro-1,1,3a,7-tetramethyl-, [1aR-(1a.α,3a.α.,7b.α)]-	C_15_H_24_	5.00 *	1.01	1.06	1.44			
	Isocaryophyllene	C_15_H_24_	1.27	2.46 × 10^7^	1.60 × 10^7^		2.22 × 10^7^	3.75 × 10^7^	
Others	Methyl salicylate	C_8_H_8_O_3_	1.08	1.19	1.35	n.i.	1.24	n.i.	BC
	(*E*)-4,8-Dimethylnona-1,3,7-triene (DMNT)	C_11_H_18_	n.i.	1.07	1.33	n.i.	1.50	3.00	FS, BC
	Acetic acid, methyl ester	C_3_H_6_O_2_	2.96 × 10^7^	2.65	3.84 × 10^6^	2.17		5.30 × 10^6^	FS
	3,5-di-*tert*-Butyl-4-hydroxybenzaldehyde	C_15_H_22_O_2_		7.08 × 10^5^	4.73 × 10^5^	1.98		1.09	
	Benzene, 1-methyl-3-(1-methylethenyl)-	C_10_H_12_	1.06	n.i.	7.46 × 10^6^	n.i.	2.46	2.06	
	Nonanal	C_9_H_18_O	1.01 *	1.07	1.39	1.07	n.i.	1.23	BC
**Bordeaux**			**PB_D3**	**PB_D5**	**FS_D3**	**FS_D5**	**BC_D3**	**BC_D4**	
Terpenoids	α-Pinene	C_10_H_16_	1.23	n.i.	2.44	4.21 *	1.01	13.3	BC
	Limonene	C_10_H_16_	1.81	1.05	n.i.	4.65	1.80	n.i.	BC
	*E*,*E* allo-Ocimene	C_10_H_16_	n.i.	1.14	2.55	1.31	1.57	2.15	
	*E*,*Z* allo-Ocimene	C_10_H_16_	n.i.	n.i.	2.22	1.08	1.1	1.22	BC
	γ-Terpinene	C_10_H_16_	15.3	2.48		n.i.	1.98	7.48 × 10^5^	
	(−)-β-Bourbonene	C_15_H_24_	1.75	5.7 *			3.34	3.62	FS
	Butanoic acid, 3-hexenyl ester, (*Z*)-	C_10_H_18_O_2_	n.i.		2.18 *	74.2	1.70 × 10^5^	12.2	FS
Others	2,6-Diisopropylnaphthalene	C_16_H_20_	2.25 × 10^6^	3.14	7.55 × 10^3^		1.21		FS
	Acetic acid, hexyl ester	C_8_H_16_O_2_	2.43	n.i.	n.i.	3.14 × 10^5^	1.12	1.11	BC
	*n*-Hexadecanoic acid	C_16_H_32_O_2_	n.i.	1.78	9.39	1.58	n.i.	1.68	

**Table 3:** MeJA- and CuSO_4_-induced VOCs. Elicitor-induced VOCs were selected at three phenological stages of the grapevines and two time points (BAG mode). VOCs were selected when FC > 1 at least 4 times out of the 6 time points (ca. > 50% of the collected samples). *: Significant *t*-test (FDR < 0.05) vs. H_2_O control samples. n.i.: not induced. Phenological stages of grapevines: pre-blossom: PB; fruit-set: FS and bunch-closure: BC. Time points: three- and five-days post treatment (D3 and D5). The VOCs in bold type were common to the 3 Bastid^®^, MeJA and CuSO_4_ treatments.

MeJA		Formula	PB_D3	PB_D5	FS_D3	FS_D5	BC_D3	BC_D5
Terpenoids	**α-Pinene**	C_10_H_16_	2.14	2.28	4.53	7.27	2.47	4.39
	**β-Pinene**	C_10_H_16_	2.82	5.00	7.07	6.76	2.78	4.83
	β-Myrcene	C_10_H_16_	2.23	1.96	9.73 *	21.8	1.65 × 10^8^ *	4.44
	α-Terpinene	C_10_H_16_	6.15	9.03 × 10^7^	38.2 *	4.77	8.17	5.43 × 10^6^
	***trans-β*-Ocimene**	C_10_H_16_	2.60	1.75	9.36	8.60	26.3	3.74
	***E,E* allo-Ocimene**	C_10_H_16_			9.97	9.99	6.68	3.81
	***E,Z* allo-Ocimene**	C_10_H_16_	2.46	3.17	10.2	11.5	28.8	4.30
	***cis*-β-Ocimene**	C_10_H_16_	2.42	1.72	3.73	5.40	8.19	1.88
	**Limonene**	C_10_H_16_	3.31	2.65	5.95	5.14	5.09	3.56
	Linalool	C_10_H_18_O	n.i.	1.18	5.71	2.75	9.56	2.27
	Humulene	C_15_H_24_	3.58	3.73	14.0	12.7	60.2 *	14.2
	β-Caryophyllene	C_15_H_24_	3.30	2.73	5.64	3.93	16.07	5.60
	**Isocaryophyllene**	C_15_H_24_	7.46		3.64 × 10^8^	1.47 × 10^8^		8.04 × 10^7^
	(1*R*,2*S*,6*S*,7*S*,8*S*)-8-Isopropyl-1-methyl-3-methylenetricyclo[4.4.0.02,7]decane-rel-	C_15_H_24_	2.05	1.07	5.98	3.55 × 10^7^	4.59 × 10^7^ *	2.38 × 10^7^
	Naphthalene, 1,2,3,5,6,8a-hexahydro-4,7-dimethyl-1-(1-methylethyl)-, (1*S*-*cis*)-	C_15_H_24_	2.08	n.i.	2.68	8.53	1.98 × 10^7^ *	2.33 × 10^7^
	α-Copaene	C_15_H_24_	1.46	1.88	1.51	2.73	117.14	6.93
	*cis*-Muurola-4(14),5-diene	C_15_H_24_	n.i.	2.14	5.76		3.50	4,47 × 10^6^
	Bicyclosesquiphellandrene	C_15_H_24_	n.i.	1.51	1.15	3.45	1,26 × 10^7^	8.54
	α-Terpineol	C_10_H_18_O	2.80	3.88 × 10^7^	33.1 *	7.27 × 10^7^	1.70 × 10^7^	1.58 × 10^7^
Others	Acetic acid	C_2_H_4_O_2_	n.i.	1.75	n.i.	1.16	1.01	1.52
	Benzene, 1,3-dimethyl-	C_8_H_10_	2.70		2.95 × 10^7^	5.78	2.36	
	α-Calacorene	C_15_H_20_	5.70	12.8	4.63	3.71 × 10^6^	9.13 × 10^5^ *	9.62 × 10^5^
	**(*E*)-4,8-Dimethylnona-1,3,7-triene (DMNT)**	C_11_H_18_	1.51	1.87	6.54 *	6.19	60.8	19.2
	Benzene, 1-methyl-3-(1-methylethenyl)-	C_10_H_12_	17.5	2.50	7.29 × 10^7^ *	15.5	35.8 *	3.20
	*o*-Cymene	C_10_H_14_	4.77	1.88	5.31 *	4.41	5.31	6.50
	Acetic acid, methyl ester	C_3_H_6_O_2_	4.45 × 10^6^	1.07	2.42 × 10^7^	n.i.		5.32 × 10^6^
	3-Hexen-1-ol, acetate, (*Z*)-	C_8_H_14_O_2_	2.68	1.05	1.22	1.45	n.i.	n.i.
	Nonanal	C_9_H_18_O	2.58	1.19	n.i.	1.44	1.37	1.11
**CuSO_4_**			**PB_D3**	**PB_D5**	**FS_D3**	**FS_D5**	**BC_D2**	**BC_D5**
Terpenoids	**α-Pinene**	C_10_H_16_	1.56	n.i.	n.i.	1.70	1.10	13.5
	**β-Pinene**	C_10_H_16_	2.00	1.10	n.i.	5.48	n.i.	9.25 × 10^5^
	***trans*-β-Ocimene**	C_10_H_16_	1.12	1.24	1.68	n.i.	1.60	1.90
	***E,E* allo-Ocimene**	C_10_H_16_	1.27	3.95	5.36	n.i.	1.68	2.19 *
	***E,Z* allo-Ocimene**	C_10_H_16_	1.33	3.88	4.97	1.02	1.60	2.28
	***cis*-β-Ocimene**	C_10_H_16_	1.31	2.35	1.29	n.i.	1.61	1.38
	**Limonene**	C_10_H_16_	1.32	1.79	n.i.	4.64	1.22	1.41
	**Isocaryophyllene**	C_15_H_24_		1.02 × 10^8^ *	7.96 × 10^4^		1.70 × 10^5^	5.37 × 10^5^
	(−)-β-Bourbonene	C_15_H_24_	1.27	12.9 *	1.54		4.68	4.37
	β-Caryophyllene	C_15_H_24_	1.35	1.60 *	n.i.	1.07	1.22	1.63 *
	Humulene	C_15_H_24_	1.89			1.39	1.28	1.53
	α-Copaene	C_15_H_24_	1.38	2.13	n.i.	n.i.	3.32 × 10^5^	1.16
	Bicyclo[5.2.0]nonane, 2-methylene-4,8,8-trimethyl-4-vinyl-	C_15_H_24_	1.42	5.84	n.i.	n.i.	1.13	1.38
	Eucalyptol	C_10_H_18_O	3.82	4.49	2.96 × 10^5^	8.25 × 10^4^		
	Butanoic acid, 3-hexenyl ester, (*Z*)-	C_10_H_18_O_2_	n.i.	3.28	1.85	n.i.	1.11 × 10^5^	10.6
Others	Benzene, 1-methyl-3-(1-methylethyl)-	C_10_H_14_	1.97 × 10^7^	1.93	1.49	3.99	6.59 × 10^4^	
	Methyl salicylate	C_8_H_8_O_3_	1.32	1.86	1.06	1.17	2.60	n.i.
	Hexanal	C_6_H_12_O	4.03 × 10^8^	2.58	2.27			3.38 × 10^5^
	*n*-Valeric acid *cis*-3-hexenyl ester	C_11_H_20_O_2_	2.94 × 10^7^	1.73 × 10^7^	n.i.		2.01 × 10^4^	1.25 × 10^6^
	**(*E*)-4,8-Dimethylnona-1,3,7-triene (DMNT)**	C_11_H_18_	5.50	2.20 × 10^8^ *	2.24	n.i.		3.37
	*n*-Hexadecanoic acid	C_16_H_32_O_2_	2.75 *	1.70	2.08	1.33	n.i.	2.16
	6*H*-Benzofuro[3,2-*c*][1]benzopyran, 3,9-dimethoxy-	C_17_H_14_O_4_	1.58 × 10^6^	1.80 × 10^6^	2.24	2.44 *	1.18	2.07

**Table 4:**
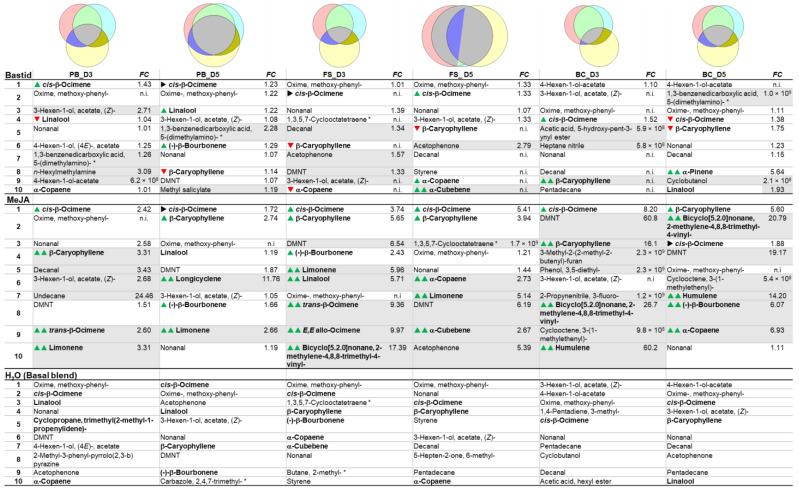
TOP10 VOCs detected in the Burgundy vineyard. Venn diagrams of the distribution of the 10 most abundant VOCs detected in Burgundy vineyard (cv. Chardonnay) for each condition and each time point (H_2_O, yellow; Bastid^®^, pink; MeJA, light blue). In the tables, compounds are ranked, from 1 to 10, according to their average intensities measured in Bastid^®^-(top table), MeJA-(middle table) and H_2_O-(bottom table) treated grapevines. The fold change (FC) relative to the H_2_O control is indicated for each induced VOC. Terpenoids are in bold type. Arrows’ variations of terpenoid ranking relative to H_2_O (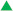 up, 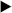 stable; 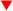 down ranking). Grey coloured lines, elicitor-induced VOCs absent in the water control; 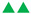 induced terpenoids. Phenological stages of grapevine: pre-blossom: PB; fruit-set: FS and bunch-closure: BC. Time points: three- and five-days post treatment (D3 and D5). n.i.: not induced; *: putative contaminant.

**Table 5:**
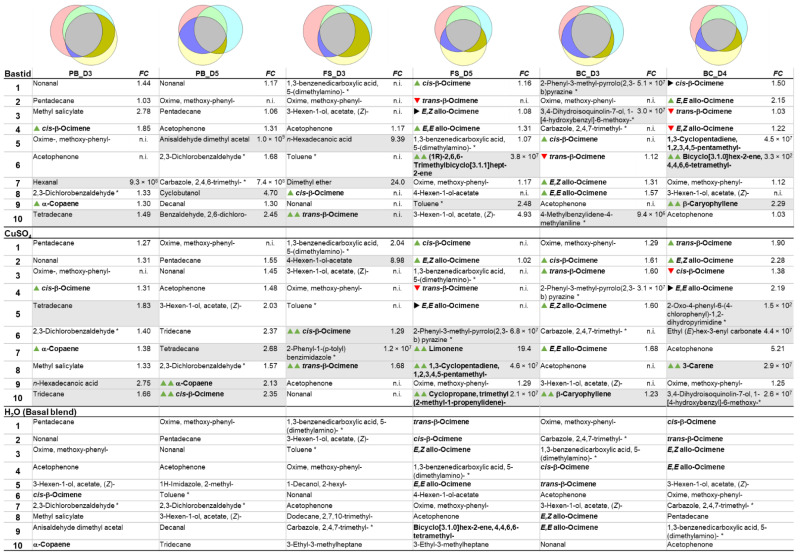
TOP10 VOCs detected in Bordeaux vineyard. Venn diagrams of the distribution of the 10 most abundant VOCs detected in Bordeaux vineyard for each condition and each time point (H_2_O, yellow; Bastid^®^, pink; CuSO_4_, light blue). In the tables, compounds are ranked from 1 to 10, according to their average intensities measured in Bastid^®^-(top table), CuSO_4_-(middle table) and H_2_O-(bottom table) treated grapevines. The fold change (FC) relative to the H_2_O control is indicated for each induced VOC. Terpenoids are in bold type. Arrows, variation of terpenoid ranking relative to H_2_O (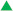 up; 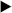 stable; 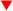 down ranking). Grey coloured lines, elicitor-induced VOCs absent in the water control; 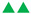 induced terpenoids. Phenological stages of grapevine: PB, pre-blossom; F, fruit-set; BC, bunch-closure. Time points: three- and five-days post treatment (D3 and D5). n.i.: not induced; *: putative contaminant.

**Table 6:** Products and doses used for the treatments in the Burgundy and Bordeaux vineyards. Dilution in water: Bastid^®^, CuSO_4_ (Bordeaux mixture) and MeJA (2.5 mM). The difference in Bastid^®^ concentrations between Burgundy and Bordeaux is due to the calibration specifications of the sprayers: a hand sprayer (600 L·ha^−1^) in Burgundy; an SR 420 atomizer (Stihl^®^, Torcy, France, 200 L·ha^−1^) in the Bordeaux vineyard.

	Bastid^®^	CuSO_4_	MeJA
**Burgundy**	50 mg/L		0.56 g/L
**Bordeaux**	156 mg/L (2.5 L/ha)	3 g/L	

## Data Availability

Not applicable.

## Supplementary Materials

The following are available online at https://www.mdpi.com/article/10.3390/molecules27186028/s1, Figure S1. Quantification of phenolics in grapevine leaves treated by Bastid^®^; Figure S2. Representative PCA illustration of VOC profile discrimination between foliage and plot edge areas; Figure S3. Impact of mean temperature and relative humidity on elicitor-induced monoterpene detection; Figure S4. Impact of mean temperature and relative humidity on elicitor-induced sesquiterpene detection; Figure S5. Impact of mean temperature and relative humidity on “Other VOCs” detections; Figure S6. Averaged Bastid vs. H2O fold changes throughout the 2019 growing season in Burgundy (BDY) and Bordeaux (BDX) vineyards; Table S1. Number of samples analysed for stilbene quantifications over 3 years (2017 to 2019) on Burgundy and Bordeaux vineyards.; Table S2. List and details of samples collected along the 3 years experiments in Burgundy (cv. Chardonnay) and Bordeaux (cv. Cabernet franc) vineyards, respectively; Table S3. List of VOC standards used in GCMS analysis.

## Sample Availability

Samples of the compounds Bastid^®^ and Bordeaux mixture are available from the authors.
