# Supplementary material for: Elicitor-Induced VOC Emission by Grapevine Leaves: Characterisation in the Vineyard
Source: Molecules. 2022 Sep 15;27(18):6028. doi: 10.3390/molecules27186028 (PMC9501231; doi:10.3390/molecules27186028)
Supplement: Supplementary file 1 [file molecules-27-06028-s001.zip › molecules-1837077-supplementary.pdf]

Supplementary Materials

Elicitor-Induced VOC Emission by Grapevine Leaves: Characterisation in the Vineyard

Christelle Lemaitre-Guillier [1,*], Agnès Chartier [2], Christelle Dufresne [2], Antonin Douillet [3], Stéphanie Cluzet [4], Josep Valls [4], Nicolas Aveline [3], Xavier Daire [1] and Marielle Adrian [1]

* Correspondence: christelle.guillier@inrae.fr

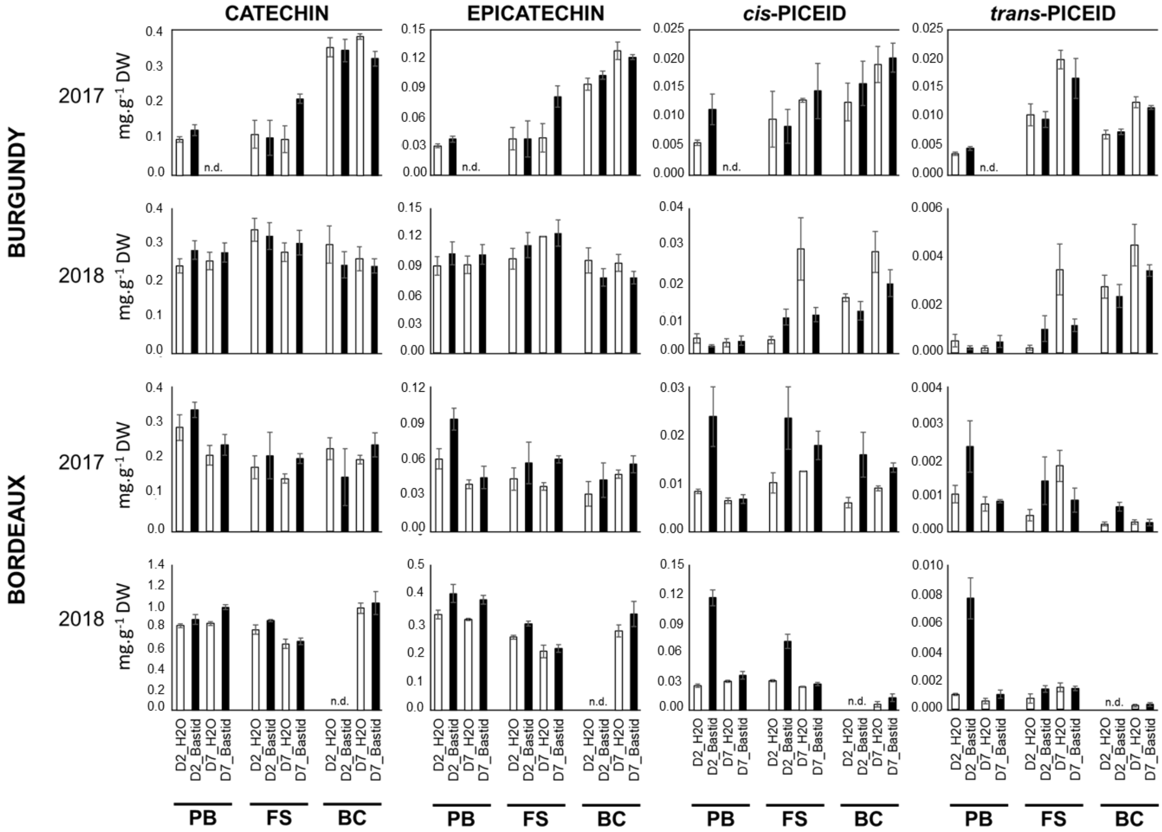

Figure S1. Quantification of phenolics in grapevine leaves treated by Bastid®. Experiments were performed in Burgundy (cv Chardonnay) and Bordeaux (cv Cabernet franc) vineyards along 3 phenological stages in 2017 and 2018. Treatments: H_2O as control (white), Bastid® (black). Catechin, epicatechin, *cis*- and *trans*-piceids were identified and quantified by LC-MS analysis. Amounts are averaged from 3 replicates and adjusted to the dry weight (DW) of green leaf powder. Phenological stages: Pre-blossom: PB, Fruit-set: FS and Bunch-closure: BC. D2 and D7: samples collected at 2 and 7 days post-treatment, respectively. n.d.: not determined.

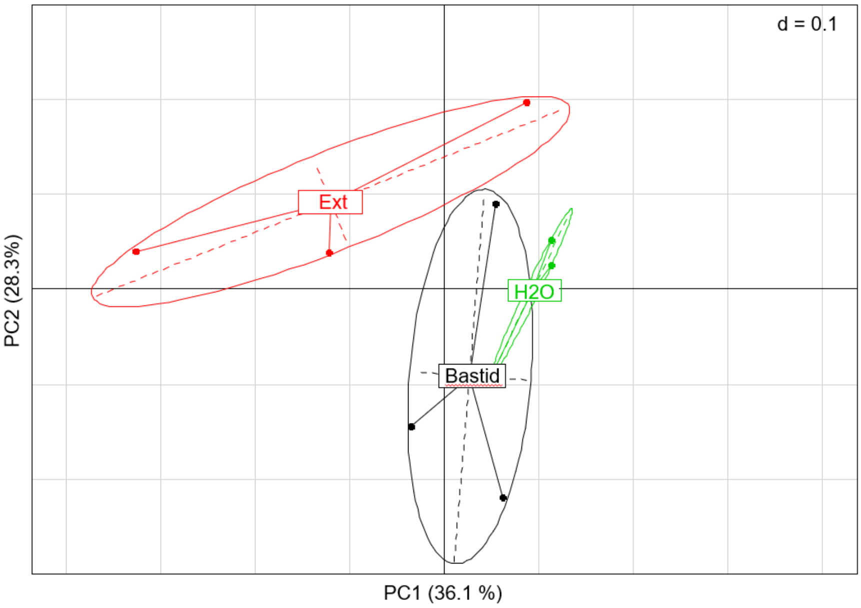

Figure S2. Representative PCA illustration of VOC profile discrimination between foliage and plot edge areas. Figure was built from 3 time points of VOCs collection in growing season 2018, at Fruit-set (FS) phenological stage, in Burgundy.

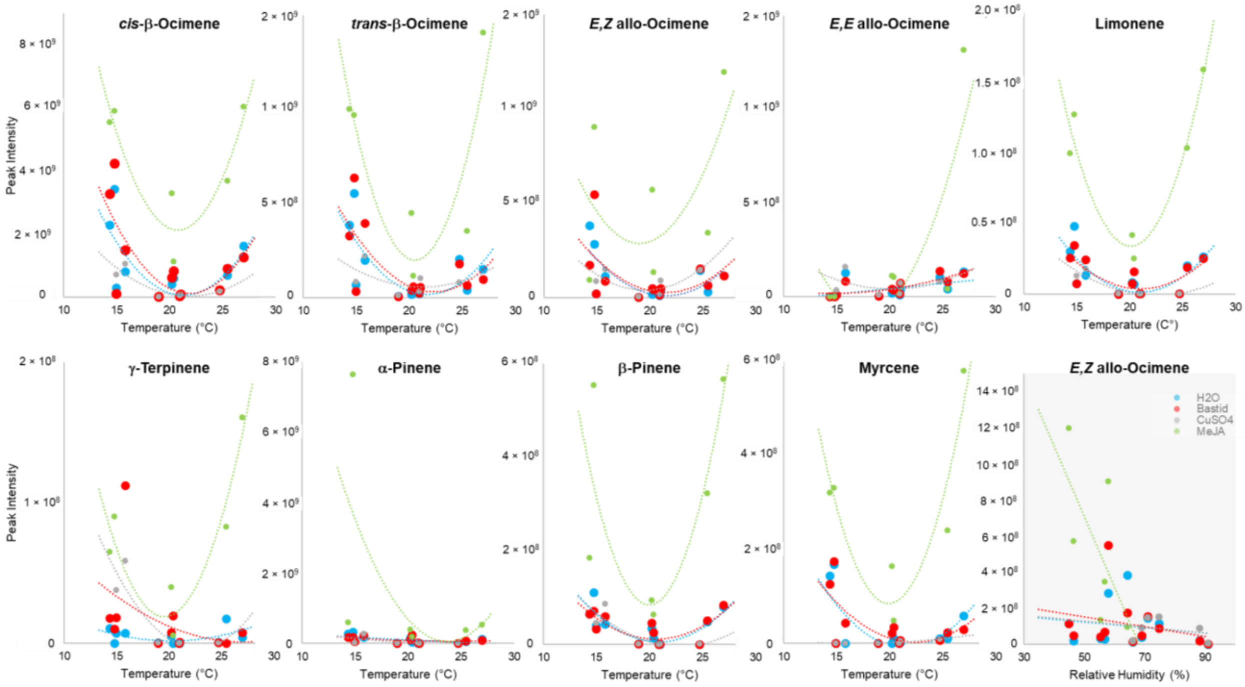

Figure S3. Impact of mean temperature and relative humidity on elicitor-induced monoterpene detection. Graphs were built from 2019 data. Grapevine emission of monoterpenes analysed at 3 and 5 days post-treatment with H_2O (as control) or elicitor (Bastid®, MeJA $CuSO_4$) at three phenological stages (Pre-blossom, Fruit-set, Bunch-closure) in Burgundy (cv. Chardonnay) and Bordeaux (cv. Cabernet franc) vineyards. Values correspond to VOC peak intensities and tendency curve shapes were chosen as the most fitting to the point values. Relative humidity graphs are represented with grey background.

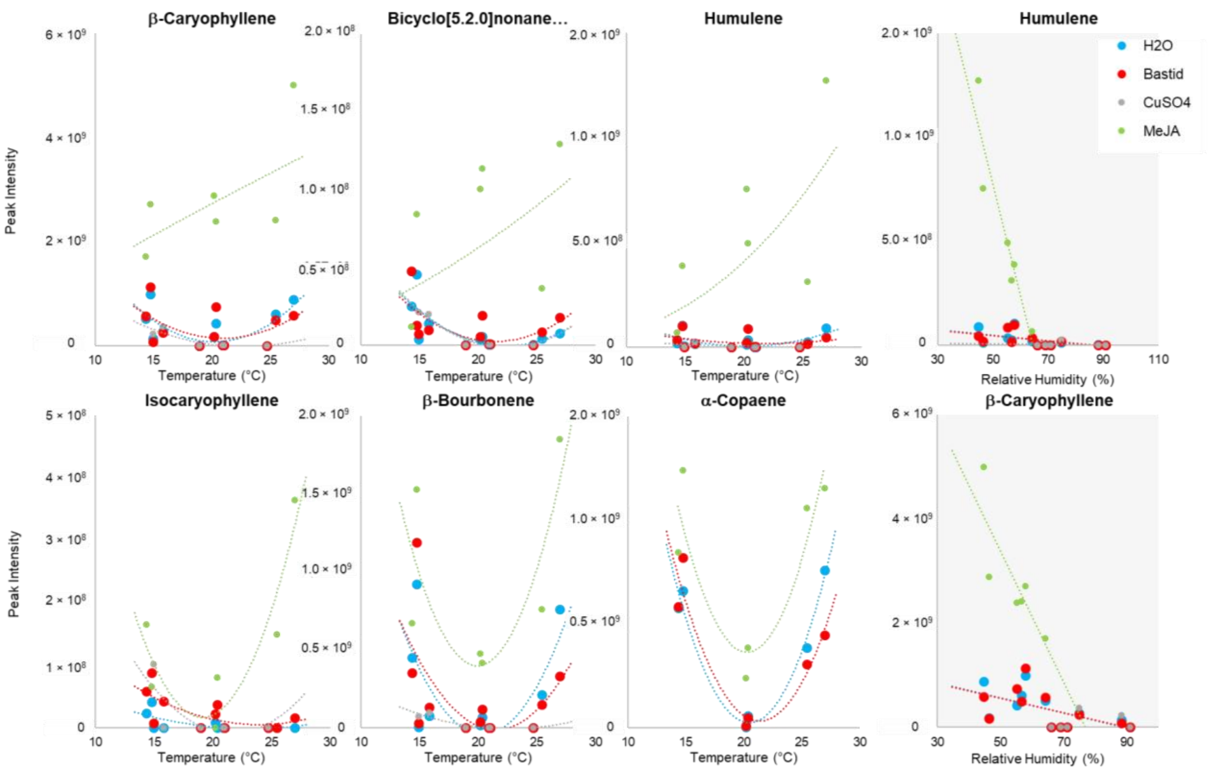

Figure S4. Impact of mean temperature and relative humidity on elicitor-induced sesquiterpene detection. Graphs were built from 2019 data. Grapevine emission of sesquiterpenes analysed at 3 and 5 days post-treatment with H_2O (as control) or elicitor (Bastid®, MeJA $CuSO_4$) at three phenological stages (Pre-blossom, Fruit-set, Bunch-closure) in Burgundy (cv. Chardonnay) and Bordeaux (cv. Cabernet franc) vineyards. Values correspond to VOC peak intensities and tendency curve shapes were chosen as the most fitting to the point values. Relative humidity graphs are represented with grey background.

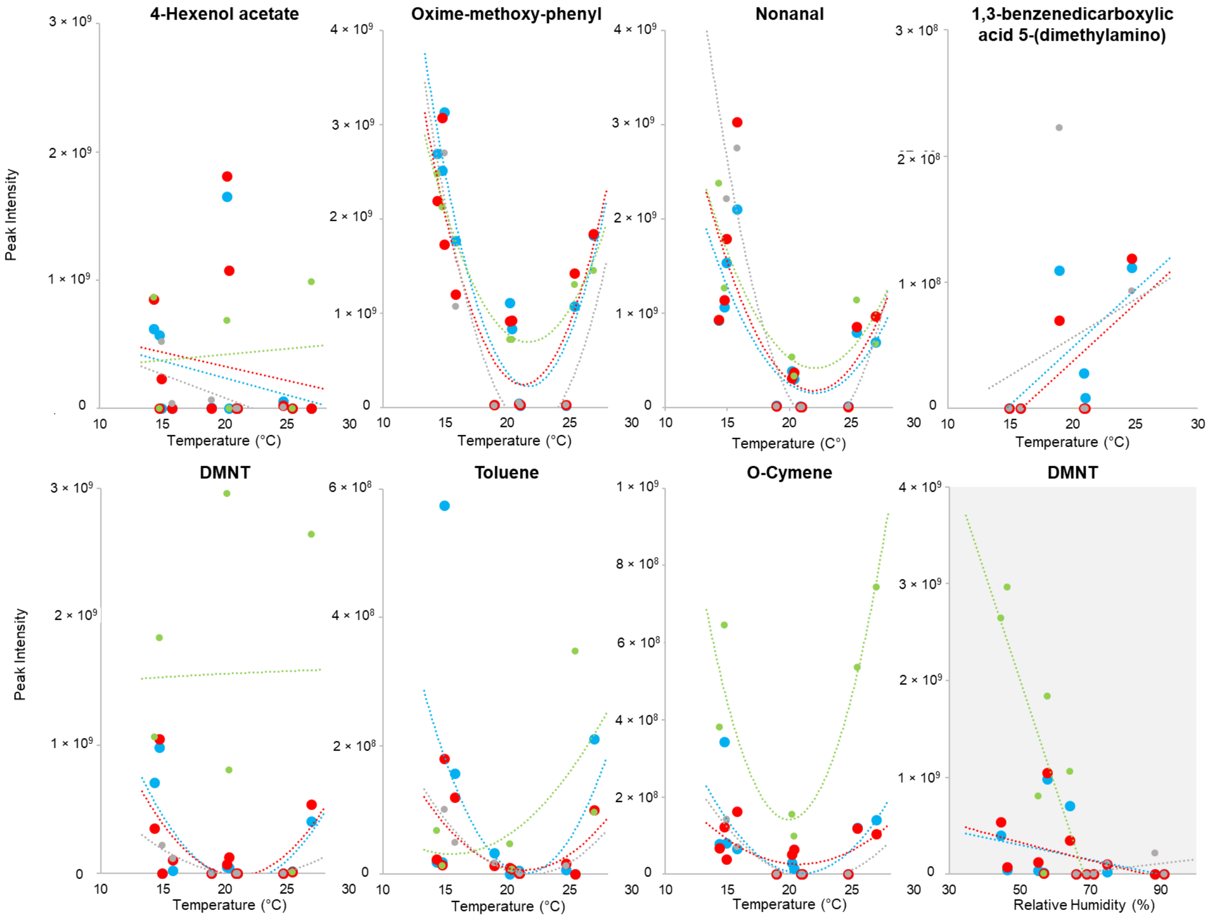

Figure S5: Impact of mean temperature and relative humidity on "Other VOCs" detections. Graphs were built from 2019 data. Grapevine emission of "Other VOCs" analysed at 3 and 5 days post treatment with H_2O (as control) or elicitor (Bastid®, MeJA $CuSO_4$) at three phenological stages (Pre-blossom, Fruit-set, Bunch-closure) in Burgundy (cv. Chardonnay) and Bordeaux (cv. Cabernet franc) vineyards. Values correspond to VOC peak intensities and tendency curve shapes were chosen as the most fitting to the point values. Relative humidity graphs are represented with grey background.

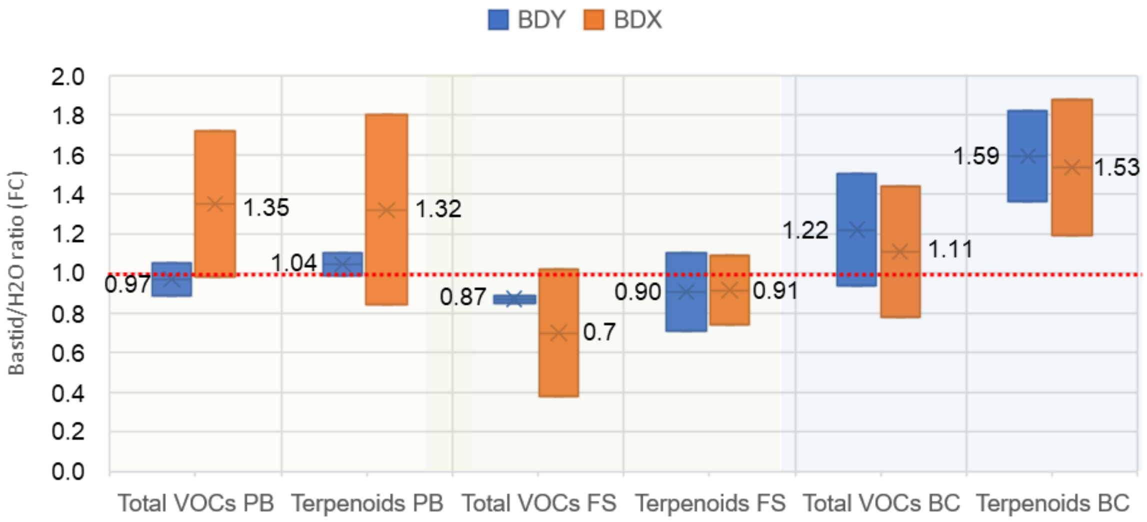

Figure S6. Averaged Bastid *vs.* H_2O fold changes throughout the 2019 growing season in Burgundy (BDY) and Bordeaux (BDX) vineyards. Each VOCs relative quantity of VOCs was averaged from replicate sample intensities at the two time points of collection per phenological stage of grapevine (pre-blossom: PB, fruit-set: FS and bunch-closure: BC) after Bastid® or H_2O treatment. Fold changes (FC) were calculated from the ratios of the Bastid®/H_2O sums of overall VOCs (Total VOCs) or terpenoids intensities.

Table S1. Number of samples analysed for stilbene quantifications over 3 years (2017 to 2019) on Burgundy and Bordeaux vineyards.

Year	2017	2018	2019
**Burgundy**	**48**	**46**	**60**
	Bastid (24), H_2O (24)	Bastid (24), H_2O (22)	Bastid (24), H_2O (24), MeJA (12)
**Bordeaux**	**48**	**48**	**72**
	Bastid (24), H_2O (24)	Bastid (24), H_2O (24)	Bastid (24), H_2O (24), $CuSO_4$ (24)

Table S2. List and details of samples collected along the 3 years experiments in Burgundy (cv Chardonnay) and Bordeaux (cv Cabernet franc) vineyards, respectively. VOCs were collected either under Open-air (OA), open air cumulated (OA-CUMUL) or bagged (BAG) modes with indicated exposure durations of collection. Samples of VOC were collected at Pre-blossom (PB), Fruit-set (FS) and Bunch-closure (BC) stages at several time-points from D0 (D2, D3, D5, D7) in 2017 and 2019 or since D0 for 3, 5 and 7 days (3D, 5D and 7D) in 2018. Total number of samples analysed by GC-MS per season are indicated in bold characters (GC-MS samples) and total number of samples per conditions are detailed in brackets.

	Year	2017	2018		2019
	**Collection mode**	**OA**	**OA_CUMUL**	**BAG**	**BAG**
	Exposure duration	24 hours	3, 5 and 7 days	4 hours	4 hours
**Burgundy (cv. Chardonnay)**	Time-points	FS: D3 D5 D7<br>BC: D2 D4	FS: 3D 5D 7D<br>BC: 3D 5D 7D	FS: D3<br>BC: D3	PB: D3 D5<br>FS: D3 D5<br>BC: D3 D5
	Conditions	Bastid (40), H_2O (40), Ext (14)	Bastid (23), H_2O (24), Ext (17)	Bastid (8), H_2O (8)	Bastid (45), MeJA (23), H_2O (45)
	Total GC-MS samples	**94**	**64**	**16**	**113**
**Bordeaux (cv. Cabernet franc)**	Time-points	FS: D3 D5 D7<br>BC: D3 D5 D7	PB: 3D 5D 7D<br>FS: 3D 5D 7D<br>BC: 3D 6D 7D	PB: D3<br>FS: D3<br>BC: D3	PB: D3 D5<br>FS: D3 D5<br>BC: D3 D4
	Conditions	Bastid (48), H_2O (48), Ext (10)	Bastid (35), H_2O (34), Ext (4)	Bastid (12), H_2O (11)	Bastid (46), H_2O (48), $CuSO_4$ (47), Ext (4)
	Total GC-MS samples	**106**	**73**	**23**	**145**

Table S3. List of VOC standards used in GCMS analysis.

**Compound Name**	**CAS#**	**Purchase reference #**
α-Pinene	80-56-8	147524
Camphene	79-92-5	C301
α-Sabinene	3387-41-5	W530597
b-Pinene	127-91-3	112089
Myrcene	123-35-3	M100005
3-Hexen-1-ol, acetate, (*Z*)-	3681-71-8	W317101
α-Phellandrene	99-83-2	W285611
3-Carene	13466-78-9	115576
R(+)Limonene	5989-27-5	183164
b-Ocimene (isomers mixture, *E*,*Z*)	13877-91-3	W353901
α-Terpinene	99-86-5	223182
γ-Terpinene	99-85-4	223190
α-Terpinolene	586-62-9	86485
Linalool	78-70-6	L2602
cis-allo-Ocimene	673-84-7	289698
Methyl salicylate	119-36-8	M6752
Longicyclene	1137-12-8	62633
b-Caryophyllene	87-44-5	22075
Humulene/α-Caryophyllene	6753-98-6	53675
Valencene	4630-07-3	W344303
(*Z*,*E*) α-Farnesene (isomers mixture)	502-61-4	W383902
Methyl Jasmonate	39924-52-2	392707
